# Single-cell analysis reveals the spatial-temporal expression of genes associated with esophageal malformations

**DOI:** 10.1038/s41598-024-53098-w

**Published:** 2024-02-14

**Authors:** Carlo Maj, Antonia Eberts, Johannes Schumacher, Pouria Dasmeh

**Affiliations:** 1 Center for Human Genetics, Marburg University and Marburg University Hospital, Marburg, Germany; 2 Department of Chemistry and Chemical Biology, Harvard University, Cambridge, USA; 3 Institute for Evolutionary Biology and Environmental Studies, University of Zurich, Zurich, Switzerland

**Keywords:** Gene expression, Cell lineage, Gastrulation

## Abstract

Understanding the molecular mechanisms of congenital diseases is challenging due to their occurrence within specific developmental stages. Esophageal malformations are examples of such conditions, characterized by abnormalities in the development of esophagus during embryogenesis. These developmental malformations encompass a range of anomalies, including esophageal atresia, and tracheoesophageal fistula. Here, we investigated the preferential expression of 29 genes that are implicated in such malformations and their immediate interactome (a total of 67 genes). We conducted our analyses across several single-cell atlases of embryonic development, encompassing approximately 150,000 cells from the mouse foregut, 180,000 cells from human embryos, and 500,000 cells from 24 human organs. Our study, spanning diverse mesodermal and endodermal cell populations and early developmental stages, shows that the genes associated with esophageal malformations show their highest cell-type specific expression in lateral plate mesoderm cells and at the developmental stage of E8.75–E9.0 days. In human embryos, these genes show a significant cell-type specific expression among subpopulations of epithelial cells, fibroblasts and progenitor cells including basal cells. Notably, members of the forkhead-box family of transcription factors, namely *FOXF1*, *FOXC1*, and *FOXD1*, as well as the SRY-box transcription factor, *SOX2*, demonstrate the most significant preferential expression in both mouse and human embryos. Overall, our findings provide insights into the temporal and cellular contexts contributing to esophageal malformations.

## Introduction

Understanding the molecular mechanisms of congenital diseases is challenging due to their occurrence primarily during a narrow developmental time window and in specific cell types^1–5^. Esophageal malformations are congenital anomalies that affect the development and structure of esophagus. These conditions, such as esophageal atresia and tracheoesophageal fistulas can lead to a spectrum of clinical complications, necessitating early detection and often surgical or medical intervention^6–8^. Investigating the molecular underpinning of esophageal malformations is important not only for enhanced diagnosis and risk assessment but also for the development of potential therapeutic strategies.

Genetic studies employing various approaches, including genome-wide association studies and exome sequencing, have identified several susceptibility genes linked to esophageal malformations^9–11^. An important question is whether these disease susceptibility genes exhibit preferential expression in particular cell types and/or at specific time points during embryonic development. Genes associated with many complex diseases show a cell type-specific expression such as neuronal/glial cells in neurodegenerative disease such as Alzheimer’s and schizophrenia^12,13^, or cardiac vascular cells in coronary artery disease^14^. However, most of these diseases manifest in adulthood, in contrast to congenital malformations that appear within a specific developmental window and in progenitor cells that later become major organs. It is within this spatial-temporal window that the genetic perturbations in susceptibility genes may disrupt the intricate interplay of cellular processes and lead to these diseases.

Here, we aim to identify and prioritize the cell types and developmental stages that most likely contribute to esophageal malformations. This prioritization serves two purposes. Firstly, it facilitates the identification of disease relevant cell types. The formation of the foregut and the subsequent organ morphogenesis during embryonic development relies on the interplay between two major populations of the definitive endoderm and splanchnic mesoderm cells^15^. We know little about whether genes associated with esophageal malformations equally affect both cell types or if one has a more prominent role in their development. Secondly, prioritizing cell types and developmental stages may help us better understand how genetic perturbations in the normal foregut development may lead to such malformations. We are particularly interested to know whether different susceptibility genes might show a preferential expression in specific cell types and at particular developmental stages.

## Results

### Genes associated with esophageal malformations

We first constructed the set of genes associated with esophageal malformations by considering the significant genes from previous GWAS studies^9^, genes that were implicated in esophageal anomalies from animal models^10^, and the genes identified from the exome analyses of patients^11^. These included 29 genes. It has been shown that in many complex diseases, genes that interact with such candidate genes are important for the disease etiology^16^. The candidate genes may directly contribute to the development of the condition, while the interacting genes could potentially affect signaling pathways or processes involved in esophageal and tracheal development^17^. We expanded our list of candidate genes by including additional 38 genes located in close proximity to our initial candidates, those co-express with them, and those whose encoded proteins physically interact with our candidates (as detailed in the Methods section). Overall, we will refer to these genes as EM (esophageal malformations)-associated genes. We used the single-cell disease relevant risk score (scDRS) method^18^ to quantify the preferential expression of EM-associated genes among different cell types and at different developmental stages. In brief, this approach compares the expression profile of a set of target genes with an equivalent number of control genes with the same average and standard deviation of expression level to those target genes. Using this approach, we aim to identify relevant cell types that show a preferential expression of EM-associated genes and investigate their expression throughout development. We use the terms disease scores and preferential expression interchangeably in this study. It's important to note that in our approach we investigate the preferential expression of genes associated with esophageal malformations in different cell clusters many of which may not satisfy the transcriptionally independent definition of different cell types^19^. For instance, they might represent a diverse set of cell types localized to a specific region in the developing embryo. Throughout this work, we use the terms cell types and cell clusters interchangeably, pointing to regional and specific cellular context at which EM associated genes are preferentially expressed.

### Associated genes with esophageal malformations are preferentially expressed in specific cell types

**Figure 1:**
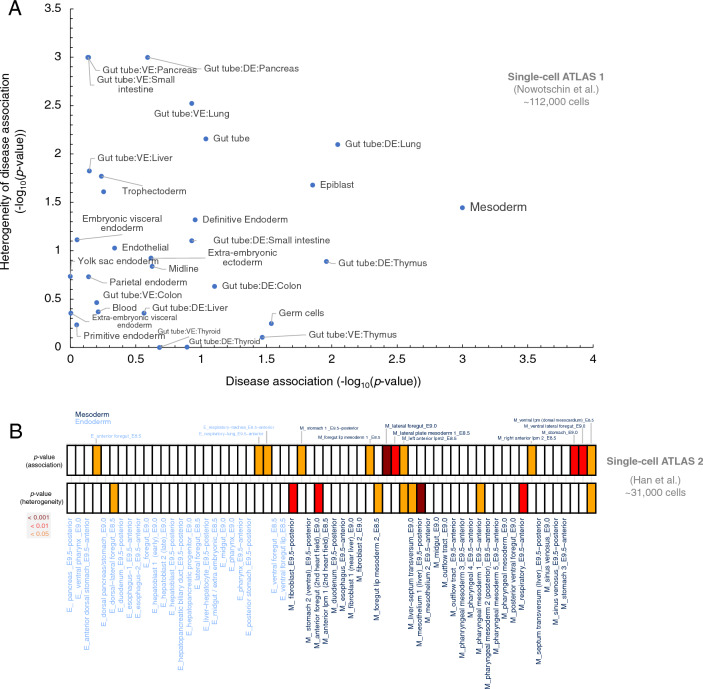
Cell Type Enrichment of genes associated with esophageal malformations. (**A**) the heterogeneity of disease association (− log_10_(*p*_heterogeneity_)) versus the disease association (-log_10_(*p*_association_)) for different cell types of the mouse gut endoderm^20^. (**B**) Enrichment of EM- associated genes in different cell types of endodermic and mesodermal origins^15^. The heatmap shows *p*-values, with colors indicating significance levels. Dark red, red, orange, and white correspond to *p*-values < 0.001, between 0.001 and 0.01, between 0.01 and 0.05, and non-significant cell types, respectively. In panel (**A**), VE and DE refers to visceral endoderm and definitive endoderm, respectively. The cell type identities in panel B are listed in Table S3. The *p*-values in all panels were calculated from the scDRS algorithm using permutation tests.

We first investigated the preferential expression of EM-associated genes among the single cells of the atlas of mouse endoderm (Nowotschin et al.)^20^, spanning from the embryonic day 3.5 (E3.5) to the embryonic day 8.75 (E8.75). We found a significant enrichment of EM-associated genes in pluripotent epiblast (*p* = 0.013), mesodermal cells (*p* = 0.00099), and the definitive endodermal cell types that descends into lung (*p* = 0.0089), and thymus (*p* = 0.010). Notably, the mesodermal cells exhibited the most significant preferential expression of EM-associated genes compared to randomly sampled control genes from the genome (Fig. 1A, Table S2). We also computed the variability in disease scores among individual cells within each cell type that measures the extent to which cell types have cells with a strong disease association, as well as non-disease associated cells. The definitive and visceral endoderm progenitor cells of the emergent organs pancreas (*p* = 0.00099) and small intestine (*p* = 0.00099) displayed the greatest heterogeneity in disease association among individual cells. This heterogeneity is characterized by some cells exhibiting a preferential expression of disease-associated genes, while others do not (Fig. 1A, Table S2).

To see the generalizability of our results, we calculated the single-cell disease scores of EM-associated genes among the cell types of the second atlas that contained 31,000 cells of developing mouse foregut, within 26 sub-clusters of definitive endoderm (E) and 36 sub-clusters of splanchnic mesoderm (M) at the three time points of E8.5, E9.0 and E9.5 (Table S3, Fig. S1)^15^. We identified a marked enrichment of our disease-relevant genes in nine mesodermal and three endodermal cell clusters (Fig. 1B, Table S3). These were cells of anterior foregut at E8.5 (E_a5; *p* = 0.042), respiratory trachea at E9.5 (E_c5; *p* = 0.029), respiratory lung at E9.5 (E_c7; *p* = 0.029), lateral plate mesoderm at E8.5 (M_a0; *p* = 0.006), foregut lip mesoderm at E8.5 (M_a1; *p* = 0.048), left anterior lateral plate mesoderm at E8.5 (M_a3; *p* = 0.012), right anterior lateral plate mesoderm at E8.5 (M_a5; *p* = 0.033), ventral lateral plate mesoderm at E8.5 (M_a6; *p* = 0.045), stomach cell types at E9.0 (M_b1; *p* = 0.006), lateral foregut at E9.0 (M_b3; *p* = 0.00099), ventral lateral foregut at E9.0 (M_b4; *p* = 0.004), and stomach at E9.5 (M_c0; *p* = 0.048). These findings further replicate our observation in the first atlas that EM-associated genes are significantly more expressed in mesodermal cell types, than cell types of endodermal origin.

### Temporal and spatial preferential expression of genes associated with esophageal malformations

**Figure 2:**
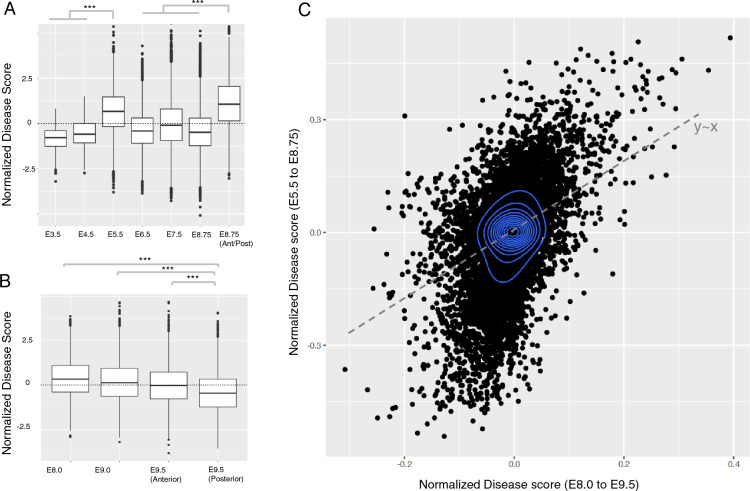
Temporal preferential expression of genes associated with esophageal malformations. (**A**) Ranked normalized disease score for all cell types within the developmental time points of E3.5, E4.5, E5.5, E6.5, E7.5, E8.75 (extracted from the descendants of either visceral or definitive endoderm in the gut tube), and E8.75 (taken from anterior/posterior halves). (**B**) Ranked normalized disease score for all cell types within the time points of E8.0, E9.0, and E9.5 in anterior and posterior regions of the foregut. Comparisons with the *p*-value < 10^–10^ from a Wilcoxon’s rank-sum test are denoted by three asterisks. (**C**) The correlation of gene expression with single-cell disease scores for 20,898 human genes in the developmental stages of E5.5–E8.75 (single-cell atlas of Nowotschin et al.^20^) (y-axis) versus the same correlation in the later stages of E8.0–E9.5 days (Han et al.^15^). The *p*-values in all panels were calculated from the scDRS algorithm using permutation tests.

We next compared the enrichment of disease-relevant genes across different developmental time points (Supplementary information, Table 1). In the first atlas^20^, the earliest time point for which EM-associated genes had preferential enrichment was E5.5 (Fig. 2A). Notably, there was no significant enrichment observed during the subsequent stages of E6.5 and E7.5 days (Tables S1, S2, Fig. 2A). However, the preferential expression of EM-associated genes at the developmental stage of E8.75 and in the cells taken from anterior/posterior ends was the most significant among all developmental stages (*p* = 0.001; scDRS permutation test). The heterogeneity of disease association was significant from E5.5 and stayed significant until E8.75. This shows that a fraction of cells that might be affected by EM-associated genes are present as early as E5.5 and also at later stages. In the second atlas^15^, EM-associated genes showed their most significant preferential enrichment at the time point of E9.0 days, compared to the control genes randomly sampled from the genome (*p* < 10^–10^, Wilcoxon rank-sum test; Fig. 2B). The disease association at the later time points, specifically at E9.5 was significantly lower compared to the earlier time points of E8.0 and E8.5 (*p* < 10^–10^, Wilcoxon rank-sum test; Fig. S3). Like our observation in the first atlas, the heterogeneity of association remained significant at all three times of E8.0, E9.0, and E9.5 (Table S3). Altogether these results show that EM-associated genes have a significantly higher preferential expression at earlier stages of development, particularly preceding the formation of major organs (Fig. 2C).

**Figure 3:**
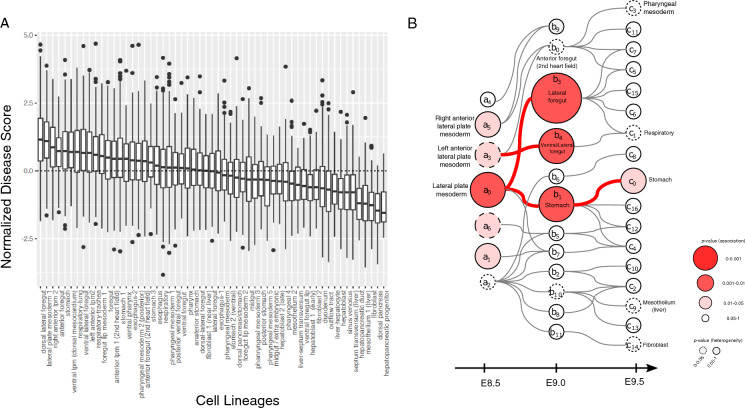
Disease scores of mesodermal cell types and cell lineages. (**A**) Normalized disease scores for distinct cell lineages of mesodermal cell types (Han et al. ^15^). (**B**) The cell fate tree of mesodermal cell types and their enrichment of EM-associated genes. The size/color of cell types reflect the significance of their association, while their dashed or solid line represent the level of heterogeneity of disease association. Gray edges represent the most likely relationship between different cell types using a single-cell voting approach^15^. Red edges connect two cell types with a significant preferential enrichment of EM-associated genes. The *p*-values in all panels were calculated from the scDRS algorithm using permutation tests.

We further looked at the disease association of mesodermal cell types throughout the development as these cell types had the highest preferential expression among all cell types of the foregut. We used both the calculated cell lineages (Fig. 3A), and the cell fate tree (Fig. 3B) of mesodermal cell types that were previously constructed by a single-cell voting approach^15^. We found that the cell types in the lateral plate mesoderm, which eventually develop into the lateral-ventral cells of the anterior foregut, show the most significant disease association. We conducted two additional analyses to validate the preferential expression of EM-associated genes in the anterior region of developing embryo. In the first analysis we compared the disease score of cells extracted from the anterior/posterior halves with the disease score of cells extracted from the descendants of either visceral or definitive endoderm (Supplementary note 1, Fig. S2). In the second analysis, we examined the expression patterns of *HOX* genes. These genes are crucial regulators of positional identity along the anterior–posterior axis during embryonic development, as well as cell-type differentiation^21–23^. We particularly the disease score of cells expressing the anteriorly expressed *HOX* genes (*HOXA1, HOXA2, HOXA3, HOXA4, HOXA5, and HOXA6*) compared to posteriorly located *HOX* gens (*HOXA9, HOXA10, HOXA11, AND HOXA13*) (Supplementary note 2, Fig. S3). Both analyses confirmed that cells at the anterior region of developing foregut have a significantly higher disease score compared to the cells located in the posterior end.

### Gene prioritization using single-cell disease scores

**Figure 4:**
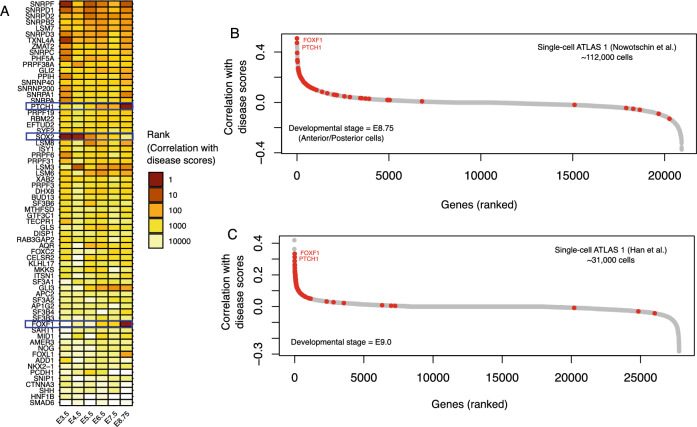
Gene prioritization using the single-cell disease scores. (**A**) The rank of EM-associated genes in their correlation with single-cell disease scores at different developmental stages of the mouse gut endoderm^20^. (**B**, **C**) The ranked correlation of the expression of 20,898 human genes with single-cell disease scores in the cells of the mouse gut endoderm (panel B, Nowotschin et al.^20^) at the time point of E8.75, and mouse foregut (Panel C, Han et al.^15^) at the time point of E9.0. The red circles represent EM-associated genes.

Next, we prioritized genes whose expression significantly influences the disease scores of individual cells. We ranked 20,898 human genes in their correlation with singe-cell disease scores in our two atlases. Notably, the ranking of EM-associated genes to disease scores varied from one developmental stage to another (Fig. 4A). We did not find any EM-associated gene that retained its position within the top 10 genes across all developmental stages suggesting that these genes might have a preferential expression during specific developmental stages. Indeed, the expression of three genes, namely *FOXF1*, *PTCH1*, and *SOX2* were the most important determinant of disease scores at different stages. The expression of SOX2 is the most important determinant of single-cell disease scores at E3.5 and this correlation decreases as the embryo transitions into later developmental stages (Figs. 4A and S4, Table S4). Conversely, *FOXF1* and *PTCH1* display a less pronounced correlation with single-cell disease scores during early development, but they undergo substantial changes and become the most prominent genes at E8.75 (Figs. 4B and S6, Table S4).

We also examined our second atlas (Han et al.^15^), particularly at the developmental stage E9.0 days, and compared the rank of human genes in disease scores with our results at E8.75 from the first atlas (Nowotschin et al.^20^). Here too, the expression of the EM-associated genes *FOXF1* and *PTCH1* showed the strongest correlation with disease scores of single cells (Fig. 4C). Both genes were the most correlated genes in our first atlas followed by the genes *PTMA*, *NASP*, *ARG1*, and *H2AFZ*. In the second atlas, the two genes *FOXF1* and *PTCH1* were the third and the fourth strongly correlated genes with disease scores across all human genes, preceded by the genes *PTMA* and *H2AFZ*. Interestingly, the genes *PTMA* and *H2AFZ* not initially considered EM- associated showed a strong correlation with disease scores. *PTMA* is involved in embryonic development and its knock down in zebrafish, results in morphogenesis defects^24^. *H2AFZ*, a member of the histone H2A family, plays a vital role in the epigenetic reprogramming of early embryonic development in mammals^25^. Our findings suggest that the genes identified in our approach but not listed within our set of disease relevant genes, may be biomarkers of foregut malformations.

### Preferential expression of genes associated with esophageal malformations in human cells

**Figure 5:**
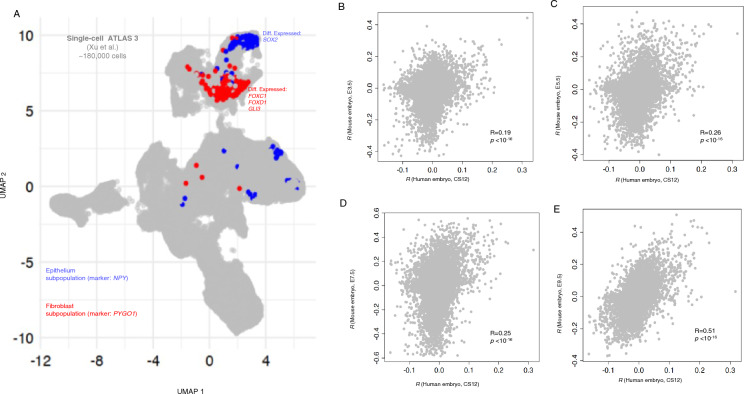
The preferential expression of genes associated with esophageal malformations in Human embryos. (**A**) The UMAP coordinates of 180 K human embryos from CS12 to CS16 from the single-cell atlas of Xu et al^26^. The cells colored in blue, and red belong to the subpopulations of epithelial cells, and fibroblasts which show the most significant preferential expression of EM associated genes among other cell clusters. (**B**–**E**) The correspondence between gene expression correlation with disease scores in human and mouse embryos for 20,897 human genes. Each gray circle shows the Pearson correlation between the gene expression in single-cells and the disease scores. The y-axis in all panels show the correlation coefficient for human genes in human embryos at the developmental stage of CS12. We compared the correlation coefficients for human genes with the correlation coefficients of mouse genes at the developmental stage of E3.5 (panel **B**), E5.5 (panel **C**), E7.5 (panel **D**), and E9.5 (panel **E**) from the atlas of Nowotschin et al.^20^.

Finally, we investigated the preferential expression of EM-association genes in human cells. We first focused on human embryos. While an exact comparison between our findings from mouse cells with human cells is not feasible due to the absence of a comprehensive single-cell dataset for human embryos, this comparative analysis is crucial. It enables us to prioritize cell clusters in human, and more specifically, to see whether similar genes contribute to the preferential expression of EM-associated genes. We used the single-cell alas of Xu et al.^26^ that comprises ~ 180,000 cells spanning the weeks 4 to 6 (Carnegie stage 12–16, CS12–CS16) obtained from aborted human embryos within 313 clusters. The developmental stage of this dataset approximately corresponds to stages E9.5–E11.5 in mouse. To align with our analysis in the mouse dataset, we calculated single-cell disease-relevant scores for all cells in this dataset, excluding neurons and progenitor neural cells. We found two clusters of cells exclusively coming from the dissected head region of the embryos incorporating trachea and esophagus that showed the most significant preferential expression of EM-associated genes (*p* ~ 0.00091, permutation test). These were clusters of epithelial cells and fibroblasts marked by the differential expression of gene markers, *NPY*, expressing neuropeptide Y, and *PYGO1* encoding for the protein Pygopus Family PHD Finger 1 (Fig. 5A, Table S5). Notably, within the fibroblast cell cluster, two members of the forkhead-box family of transcription factors, namely FOXC1 and FOXD1, were differential expressed^26^. Additionally, we identified SOX2 as a shared gene between EM-associated genes and the differentially expressed genes within the epithelial subpopulation^26^.

We further conducted a comparative analysis comparing gene expression correlation with disease scores in both human and mouse embryos for 15,209 genes. Specifically, we assessed the Pearson correlation between gene expression in single cells and disease scores across four developmental stages of E3.5, E5.5, E7.5, and E9.5 in mouse (from the atlas of Nowotschin et al.^20^) with the developmental stage of CS12 in human (the Fig. 5B–E). The developmental stage CS12 in humans approximately aligns with E9.5 in mice. We anticipated that, as the mouse developmental stage approached its corresponding phase in humans, we would observe an improved alignment between gene expression and disease scores. Indeed, as shown in Figures B–E, we observed a strengthened correlation between gene expression and disease scores, especially with the increasing alignment between mouse and human time points (*p* < 0.001, Fisher Z-transformation, Supplementary note 3). This underscores the significance of temporal alignment in the association between gene expression and disease scores in both species.

To investigate the potential persistence of preferential expression of esophageal malformation (EM)-associated genes beyond the embryonic phase and into adult tissues, we extended our analysis to include the expression patterns across approximately 500,000 cells within the Tabula Sapiens human single-cell atlas, encompassing cell types from 24 different organs^27^. Our analysis in mouse embryos indicated a heightened preferential expression of EM-associated genes in progenitor cells, and we investigated whether a similar trend might be observed in humans as human adult tissues also harbor a reservoir of progenitor cells. Remarkably, the most significant cell type that exhibited a preferential expression of EM-associated genes were myofibroblast cells in the bladder and adipose tissues, followed by basal cells in the combined set of epithelial cells from all tissues as well as the basal cells of the lung (Table S5). Given that basal cells serve as progenitors of the airway epithelium, genetic disruptions in EM-associated genes could potentially influence the replenishment of epithelial cells in line with previous clinical observations that patients with esophageal malformations often experience respiratory conditions^28^. These results also suggest that the analysis of cell type-specific expression of disease relevant genes may provide further insights into other comorbidities that are frequently observed in congenital diseases.

## Discussion

Altogether, our findings demonstrate that genes associated with esophageal malformations are preferentially expressed at the developmental stage of ~ E8.75–E9.0 days, and within mesodermal cell types in mouse, particularly lateral plate mesoderm cells. In human embryos, this preferential expression within the developmental stages of CS12–CS16, mainly occurs in the subpopulations of epithelial cells and fibroblasts. The early preferential expression of *SOX2* among EM-associated genes is in line with previous observation that an altered expression of *SOX2* impacts dorsal/ventral patterning in the anterior foregut^29^. Within human embryos, *SOX2* was also the most preferentially expressed gene among our set of EM-associated genes at all the three developmental stages of CS12, CS13-14, and CS15-16. We also observed a co-expression of *FOXF1* and *PTCH1* in mouse embryos in mesodermal cell types which highlights the involvement of the Sonic hedgehog (Shh) signaling pathways. Activation of the Shh pathway occurs through PTCH1 inhibition on Smoothened (SMO), leading to subsequent GLI activation and *FOXF1* expression. Additionally, and approximately around the E8.75–E9.0 days, *FOXF1* and *PTCH1* emerge as the most significant preferentially expressed genes, aligning with *FOXF1*-mediated mesoderm thickening, septum formation, and tracheoesophageal separation^30^, processes frequently implicated in esophageal atresia. 

How do our results align with the observed differentially expressed genes, pathways, and affected cell types in individuals with esophageal malformations? A notable challenge in this comparison arises from the scarcity of gene expression data in patients with these malformations during prenatal and embryonic stages. Nevertheless, there are a few studies that have examined the transcriptomic patterns of individuals with esophageal malformations post-birth^31–35^. The recent work by Brosens et al.^35^ provides a comprehensive analysis of the whole-genome transcription profiling and immunohistochemistry of tissue samples from patients with tracheoesophageal fistulas, that had undergone surgery in 2–16 days after birth. Our results align with several observations in this study. Firstly, the key genes such as the members of the forkhead-box family of transcription factors including *FOXF1*, *FOXC1*, and *FOXD1* as well as the gene *PTCH1* are upregulated in patients with tracheoesophageal fistulas compared to either lung, trachea or esophagus controls. These are also the genes with the highest preferential expression in single cells (Fig. 4B, C). Secondly, the case of SOX2 is notwithstanding. Brosens et al. noted a distinctive cytoplasmic staining pattern of SOX2 in control samples from both the esophagus and trachea. In contrast, patients with tracheoesophageal fistulas exhibited a noticeable shift, with SOX2 displaying clear nuclear labeling in epithelial cells. The intriguing possibility that the reduced expression of SOX2 is associated with this altered cellular localization and whether such association is cell-type specific raises question for future investigations given that similar changes in SOX2 have been also reported in esophageal squamous cell carcinoma^36^. Thirdly, cell types that are normally present in esophagus are also present in patients with TEF albeit with disorganized cell layers implying that the etiology of esophageal malformations such as TEF are likely in processes involved in anterior–posterior or dorsal–ventral axis patterning^35^. This observation aligns with our findings that genes associated with esophageal malformations have a significantly higher disease relevance at earlier developmental stages during the mouse foregut development (Figs. 2 and 3) and particularly in lateral plate mesoderm cells (Fig. 3B).

It is crucial to acknowledge two key limitations in our study. First, our focus primarily centered on genes implicated in esophageal malformations through genetic analysis methods, relying on data from genome-wide association studies, exome sequencing, and mutational studies in animal models. Including genes that are differentially expressed in both patients with esophageal malformations and animal models would offer a more comprehensive perspective on how genetic perturbations contribute to these malformations. Second, we should stress that our findings best serve as providing testable biological hypotheses and future experimental validations are necessary to prove the involvement of our identified cell clusters and prioritized genes at the molecular level before claiming the identification of casually associated genes.

Lastly, we anticipate that future studies employing single-cell disease scores for other congenital diseases and developmental anomalies could reveal spatial-temporal associations that are often obscured by the absence of single-cell resolution. We also anticipate that extensive longitudinal data, obtained through expression profiling at distinct time points or transcriptome-wide association studies focused on specific developmental stages, will aid in prioritizing disease-relevant genes and cell types, providing a deeper understanding of the mechanisms underlying the pathogenesis of congenital diseases.

## Methods

### Compiling the list of associated genes with esophageal malformations

We first constructed the set of EM-associated genes by concatenating the significant genes from our previously published GWAS study^9^, namely CTNNA3, FOXF1, FOXC2, FOXL1, HNF1B, genes that were implicated in esophageal anomalies^10^, including MTHFSD, MID1, MKKS, SHH, GLI2, GLI3, NOGGIN, NKX2-1, EFTUD2, SOX2, ADD1, GLS, AP1G2, TECPR1, KLHL17, CELSR2, DISP1, SMAD6, as well as genes identified from an exome analysis patients with esophageal malformations^11^, namely APC2, AMER3, PCDH1, GTF3C1, RAB3GAP2, and ITSN1 (For the full annotation, and biological functions of these genes check Table S1). We refer to these 29 genes as our primary set of EM-associated genes. We selected genes that interact with our primary list of EM-associated genes from their interaction scores in the STRING database^29^. Particularly, we set the maximum number of interactors to be 20 genes both in the first and the second interaction shell and identified 38 genes that interact with our primary set of disease-associated genes. We observed 79 interactions with high confidence (STRING score > 0.7) among the combined sets of disease-associated genes (PPI enrichment *p*-value < 10^–16^). The functional enrichment of different ontology terms show that EM-associated genes are enriched in several related phenotypes such as dorsal–ventral pattern formation (*p*-value ~ 10^–7^), embryonic digestive tract development (*p*-value ~ 10^–7^), as well as biological processes related to mRNA splicing (*p*-value ~ 10^–57^) (Table S1).

### Single-cell datasets and single-cell disease relevant scores

Here, we investigated the preferential expression of EM-associated genes across a diverse spectrum of cell types and throughout the embryonic development. We analyzed two single-cell atlases of mouse embryonic development. The first atlas referenced as Nowotschin et al.^20^ comprises of ~ 112,000 cells across 30 different cell types of mouse endoderm, spanning from embryonic day 3.5 (E3.5) to embryonic day 8.75 (E8.75). The second atlas from Han et al.^15^ comprises of 31,000 cells within 26 sub-clusters of definitive endoderm (E) and 36 sub-clusters of splanchnic mesoderm (M) at three time points of E8.5, E9.0 and E9.5 during mouse foregut development. For the adult human cell types, we analyzed the expression of EM-associated genes within ~ 500 K cells of 24 organs within the single-cell atlas of Tabula Sapiens^27^.

We employed the single-cell disease relevant risk score (scDRS) methodology, a computational approach designed to quantify the disease relevance of individual cells based on their gene expression profiles. scDRS compares the expression level of disease relevant genes with an equivalent number of control genes with the same average and standard deviation of expression level to those of disease relevant genes^18^. Based on this comparison, a disease score is assigned to each single-cell if it exhibits a significantly higher expression of disease-related genes. We used the EM-associated gene set and the single-cell count matrices of the two atlases (in h5ad format) as inputs and calculated the disease relevant scores using the “compute_score” function. We conducted 1,000 permutations of a control gene set. Each permutation consisted of randomly selecting 1,000 genes with comparable average and standard deviation of expression levels to our set of disease-associated genes.

We employed Scanpy (v1.9.3) within Python (v3) to process single-cell transcriptomics data. For all comparative analyses, expression values were log-transformed using a scaling factor of 10,000. To test the null hypothesis that correlations are identical between mouse and human data (Fig. 5B–E), we used Fisher’s z-transformation. In this statistical method, Pearson’s or Spearman's correlation coefficients are converted to z-scores, so that they become normally distributed (Supplementary note 3). The null hypothesis is then tested using a t-test on the z-scores. Statistical analyses were conducted using R (v4.2.1).

### Supplementary Information

Supplementary Information 1.

Supplementary Information 2.

Supplementary Information 3.

Supplementary Information 4.

Supplementary Information 5.

Supplementary Information 6.

## Data Availability

The datasets analyzed in this study and the corresponding scripts can be accessed through our GitHub repository: https://github.com/dasmeh/Foregut_malformations.
