# Supplementary material for: Single-cell analysis reveals the spatial-temporal expression of genes associated with esophageal malformations
Source: Sci Rep. 2024 Feb 14;14:3752. doi: 10.1038/s41598-024-53098-w (PMC10866870; doi:10.1038/s41598-024-53098-w)
Supplement: Supplementary file 1 — Supplementary Information 1. [file 41598_2024_53098_MOESM1_ESM.docx]

**Supplementary Information**

**Single-cell Analysis Reveals the Spatial-temporal Expression of Genes Associated with Esophageal Malformations**

Carlo Maj^1^*,^ Antonia Eberts^1^^, Johannes Schumacher^1#^*, Pouria Dasmeh^1,2,3#^*

^1^Center for Human Genetics, Marburg University and Marburg University Hospital. ^2^Department of Chemistry and Chemical Biology, Harvard University. ^3^Institute for Evolutionary Biology and Environmental Studies, University of Zurich.

^^, #^ Equal contribution

*Corresponding Authors: [Carlo.maj@uni-marburg.de](mailto:Carlo.maj@uni-marburg.de); [Dasmeh@fas.harvard.edu](mailto:Dasmeh@fas.harvard.edu)

**Figure S1.** Normalized disease scores for distinct cell types of mesodermal (M_) and endodermal (E_) origins (Table S3). Cell types with significantly higher or lower disease scores than zero are highlighted in red and blue, respectively. We used a two-sample t-test to compute p-values (adjusted for multiple sampling by Bonferroni correction) for cell types with scores greater or smaller than zero. Single-cell gene expression data is taken from the second atlas used in our study (Han et al.)^1^.

**Figure S2.** Density distribution of normalized disease scores is shown for single-cell populations at E8.75, extracted from the anterior/posterior halves (red), and from gut tube progenitor cell types (blue). Data is taken from the single-cell atlas of (Nowotschin et al.)^2^.

**Figure S3.** The normalized disease score of individual cells is plotted against the ratio of the expression of anteriorly expressed Hox genes (HOX_1-6_) to that of posteriorly expressed Hox genes (HOX*_9-13_*). The correlation coefficient denotes Spearman’s rank correlation. Gene expression data is taken from cells of the mouse endoderm atlas (Nowotschin et al.)^2^.

**Figure S4.** The rank of different genes in their expression correlation with single-cell disease scores at different stages of development from E3.5 to E8.75. Data is taken from the single-cell atlas of Han et al. ^1^

**Supplementary Table 1.** The significance for the preferential enrichment of EM-associated genes across different developmental stages and their heterogeneity. The p-values in all panels were calculated from the scDRS algorithm using permutation tests (Methods).

|  | **Developmental Stage** | **Number of cells** | ***p*-value (association)** | ***p*-value (heterogeneity)** |
| --- | --- | --- | --- | --- |
| **ATLAS 1** (*Nowotschin et al.*) | E3.5 | 774 | 0.8931 | 0.2448 |
|  | E4.5 | 232 | 0.8611 | 0.3766 |
|  | E5.5 | 11550 | 0.0250 | 0.0030 |
|  | E6.5 | 9244 | 0.5914 | 0.0200 |
|  | E7.5 | 33850 | 0.0929 | 0.0150 |
|  | E8.75 | 42599 | 0.5195 | 0.0440 |
|  | E8.75  (Anterior/ Posterior) | 5423 | 0.0010 | 0.0010 |
| **ATLAS 2**  (*Han et al.*) | E8.5 | 2182 | 0.0829 | 0.0679 |
|  | E9.0 | 3940 | 0.0270 | 0.0300 |
|  | E9.5 (Anterior) | 4580 | 0.2368 | 0.0070 |
|  | E9.5 (Posterior) | 3843 | 0.6044 | 0.0350 |

**Supplementary Note 1**

To further elaborate on this observation, we compared the disease scores of two sets of cell populations at E8.75 from the atlas of Nowotschin et al.^2^: cells extracted from the anterior/posterior halves and pooled into one cell population (E8.75_ap), and the cells extracted from the descendants of either visceral or definitive endoderm using GFP markers and fluorescent activated cell sorting (E8.75_vd). Interestingly, single-cell disease scores of EM-associated genes were significantly higher in the cells located in anterior/posterior regions (E8.75_ap) compared to the visceral and definitive endoderm descendant cell types within the gut tube (*p*<10^-16^, Kolmogorov-Smirnov test). This shows that cell types of the splanchnic mesoderm and definitive endoderm which arise at earlier stages of embryonic development and are located at either anterior or posterior locations are more important than the progenitor cells of different organs.

**Supplementary Note 2**

To further clarify the anterior/posterior relevance of cell types with high disease scores, we examined the expression patterns of HOX genes. HOX genes are pivotal in shaping the developmental axial patterns, helping to define the body plan. Importantly, the initial six HOX genes *HOXA1*, *HOXA2*, *HOXA3*, *HOXA4*, *HOXA5*, and *HOXA6* form a gene cluster that is predominantly expressed in the anterior region of the embryo^8^, compared to the HOX genes *HOXA9, HOXA10, HOXA11, AND HOXA13* that are more likely expressed in the posterior region. Indeed, cells with higher disease scores were more likely expressed in the anterior end (*R*=0.34, *p*=10^-16^, Spearman’s rank correlation). These observations highlight that susceptibility genes are preferentially expressed in the anterior region of the developing embryo, suggesting that FM likely manifests itself around the gastrulation stage and when the differences between the anterior and posterior axes become more pronounced.

**Supplementary Note 3**

To find out whether a change in correlation between two quantities is significant, we used Fisher’s Z-test. To this end, we first converted two Spearman correlation coefficients *R*_1_ and *R*_2_ into the z-scores z_1_ and z_2_:

$z_{1}=0.5\ln\left( \frac{1+R_{1}}{1-R_{1}} \right)$ (Eq. 1)

$z_{2}=0.5\ln\left( \frac{1+R_{2}}{1-R_{2}} \right)$ (Eq. 2)

We then calculated the standard errors of the z-scores, s_1_ and s_2_, as

$s_{1}=\frac{1}{\sqrt{N_{1}-2}}$ (Eq. 3)

$s_{2}=\frac{1}{\sqrt{N_{2}-2}}$ (Eq. 4)

In equations 3 and 4, we subtracted 2 from the sample size because two degrees of freedom are lost when we estimate population parameters from the sample, one for each of the two variables involved. We then calculated the test statistic Z using the following formula:

$Z=\frac{z_{1}-z_{2}}{\sqrt{{s_{1}}^{2}+{s_{2}}^{2}}}$, (Eq. 5)

and determined the corresponding p-value as:

$p=2*(1-pnorm(\left| Z \right|)$ (Eq. 6)

Here, $pnorm(\left| Z \right|)$ represents the cumulative distribution function (CDF) of the standard normal distribution (with mean zero and variance 1) for the absolute value |Z| of the test statistic, *Z*.

References

1. Han, L. *et al.* Single cell transcriptomics identifies a signaling network coordinating endoderm and mesoderm diversification during foregut organogenesis. *Nature communications* **11,** 4158; 10.1038/s41467-020-17968-x (2020).

2. Nowotschin, S. *et al.* The emergent landscape of the mouse gut endoderm at single-cell resolution. *Nature* **569,** 361–367; 10.1038/s41586-019-1127-1 (2019).

3. Gehlen, J. *et al.* First genome-wide association study of esophageal atresia identifies three genetic risk loci at CTNNA3, FOXF1/FOXC2/FOXL1, and HNF1B. *HGG advances* **3,** 100093; 10.1016/j.xhgg.2022.100093 (2022).

4. Shaw-Smith, C. Genetic factors in esophageal atresia, tracheo-esophageal fistula and the VACTERL association: roles for FOXF1 and the 16q24.1 FOX transcription factor gene cluster, and review of the literature. *European journal of medical genetics* **53,** 6–13; 10.1016/j.ejmg.2009.10.001 (2010).

5. Wang, J. *et al.* Novel candidate genes in esophageal atresia/tracheoesophageal fistula identified by exome sequencing. *European journal of human genetics : EJHG* **29,** 122–130; 10.1038/s41431-020-0680-2 (2021).

6. Barrio-Hernandez, I. *et al.* Network expansion of genetic associations defines a pleiotropy map of human cell biology. *Nature genetics* **55,** 389–398; 10.1038/s41588-023-01327-9 (2023).

7. Mering, C. von *et al.* STRING: a database of predicted functional associations between proteins. *Nucleic acids research* **31,** 258–261; 10.1093/nar/gkg034 (2003).

8. Pearson, J. C., Lemons, D. & McGinnis, W. Modulating Hox gene functions during animal body patterning. *Nature reviews. Genetics* **6,** 893–904; 10.1038/nrg1726 (2005).
